# Giant Kimura Disease of the Parotid Region Managed by Modified Rhytidectomy: A Case Report and Review of Surgical Treatment Strategies

**DOI:** 10.7759/cureus.87498

**Published:** 2025-07-08

**Authors:** Tatsuya Ishii, Naoki Matsuura, Edward H Ntege, Tomoe Sunakawa, Yusuke Shimizu

**Affiliations:** 1 Plastic and Reconstructive Surgery, University of the Ryukyus Hospital, Ginowan, JPN; 2 Pathology, University of the Ryukyus Hospital, Ginowan, JPN

**Keywords:** case report, kimura disease, modified rhytidectomy, parotid region, surgical management

## Abstract

Kimura disease (KD) is a rare, chronic inflammatory disorder that typically presents as a painless mass in the head and neck region. It is histologically characterized by lymphoid follicular hyperplasia, eosinophilic infiltration, and vascular proliferation. We report the case of a 66-year-old Japanese man with an 18-year history of recurrent, treatment-resistant KD involving the right parotid region. He presented with a large (9×6.5 cm), well-defined, firm, immobile mass that caused significant facial asymmetry and earlobe ptosis. Surgical excision was performed using a modified rhytidectomy incision, which provided wide exposure and allowed for complete removal with minimal visible scarring. A superficial parotidectomy was performed without elevating the superficial musculoaponeurotic system layer, thus preserving postoperative facial symmetry. Histopathologic examination confirmed KD, revealing follicular hyperplasia with dense eosinophilic infiltration, consistent with the hallmark features of the disease. Diffusion-weighted imaging demonstrated a hyperintense lesion with no evidence of necrosis or invasive characteristics. The patient had an uneventful postoperative course and retained House-Brackmann Grade I facial nerve function. At his six-month follow-up, he exhibited restored facial symmetry, inconspicuous scarring, and no clinical or radiologic evidence of recurrence. This case highlights the importance of integrating aesthetic surgical principles into the management of benign inflammatory lesions in the parotid region, achieving excellent functional and cosmetic outcomes. Long-term surveillance remains ongoing to monitor for potential recurrence.

## Introduction

Kimura disease (KD) is a rare, chronic inflammatory disorder that most often presents as a painless, slow-growing, superficial soft tissue mass in the head and neck region. Histologically, KD is characterized by florid lymphoid follicular hyperplasia, marked eosinophilic infiltration (often forming microabscesses), and prominent proliferation of postcapillary venules. KD predominantly affects young to middle-aged Asian men, with a reported male-to-female ratio of approximately 4:1. However, increasing reports in non-Asian populations suggest growing global recognition and diagnostic awareness [1-3].

The precise prevalence of KD remains uncertain. A 2018 review by Li et al. [4] estimated that only 200 cases had been reported in the English-language literature at that time, underscoring the condition's rarity. First described by Kim in 1937 and subsequently delineated by Kimura et al. in 1948, KD continues to pose diagnostic and therapeutic challenges due to overlapping features with both benign and malignant lesions [1,2].

Although the etiology of KD is unclear, it is believed to represent a chronic allergic or autoimmune reaction triggered by environmental or infectious antigens. Immunologically, KD exhibits a T-helper type 2 (Th2)-dominant immune response characterized by elevated interleukin (IL)-4, IL-5, and immunoglobulin E (IgE), accompanied by peripheral eosinophilia [4-6]. Recent studies also suggest the involvement of IL-21 and activated kinase pathways, indicating broader immune activation [6]. Systemic manifestations, particularly renal involvement such as nephrotic syndrome, occur in approximately 12-20% of patients with KD and are thought to result from IgE-mediated immune complex deposition and eosinophilic infiltration of the glomeruli [7].

Clinically, KD may mimic a variety of other head and neck pathologies, especially benign parotid neoplasms such as Warthin's tumor or pleomorphic adenoma. It also shares features with angiolymphoid hyperplasia with eosinophilia (ALHE), which typically presents as superficial dermal nodules without florid follicular hyperplasia, and IgG4-related disease, which is commonly associated with storiform fibrosis and obliterative phlebitis. Radiologically, contrast-enhanced magnetic resonance imaging (MRI) and diffusion-weighted imaging (DWI) are helpful in delineating lesion boundaries, typically showing iso- to hyperintense masses without necrosis or invasive characteristics [8]. Although fine-needle aspiration cytology (FNAC) may raise suspicion, its inability to preserve architectural features makes it insufficient for definitive diagnosis, necessitating excisional biopsy for confirmation [9].

Surgical excision remains the mainstay of treatment for localized KD, especially when mass effect or cosmetic concerns are present. However, complete resection can be challenging due to the lesion's ill-defined microscopic borders, and recurrence rates range from 25% to 30% [3,5]. Given KD's location in cosmetically sensitive regions, aesthetic surgical planning is essential to balance effective resection with contour preservation. Adjunctive treatments, including systemic corticosteroids, immunosuppressants, radiation therapy, and biologic agents, have shown variable success in preventing recurrence [10].

Here, we report the case of a giant KD lesion in the parotid region treated using a modified rhytidectomy (facelift-type) incision. Giant KD lesions are rare and present distinct surgical challenges due to facial distortion, subcutaneous tissue displacement, and proximity to vital structures. This novel approach enabled complete resection while preserving facial nerve function and achieving excellent aesthetic outcomes. The case underscores the value of integrating aesthetic surgical techniques into the management of chronic inflammatory masses located in cosmetically sensitive regions.

## Case presentation

A 66-year-old Japanese man presented with a progressively enlarging mass in the right parotid region. His medical history included surgical resection of a histologically confirmed KD lesion from the left parotid gland in 2005. Although detailed surgical records from that time are unavailable, no complications or postoperative sequelae were reported. He remained disease-free for over a decade but began to notice gradual swelling and increasing facial asymmetry over the preceding two years. He reported no pain, systemic symptoms, fever, or prior medical history suggestive of renal dysfunction or nephrotic syndrome.

**Figure 1:**
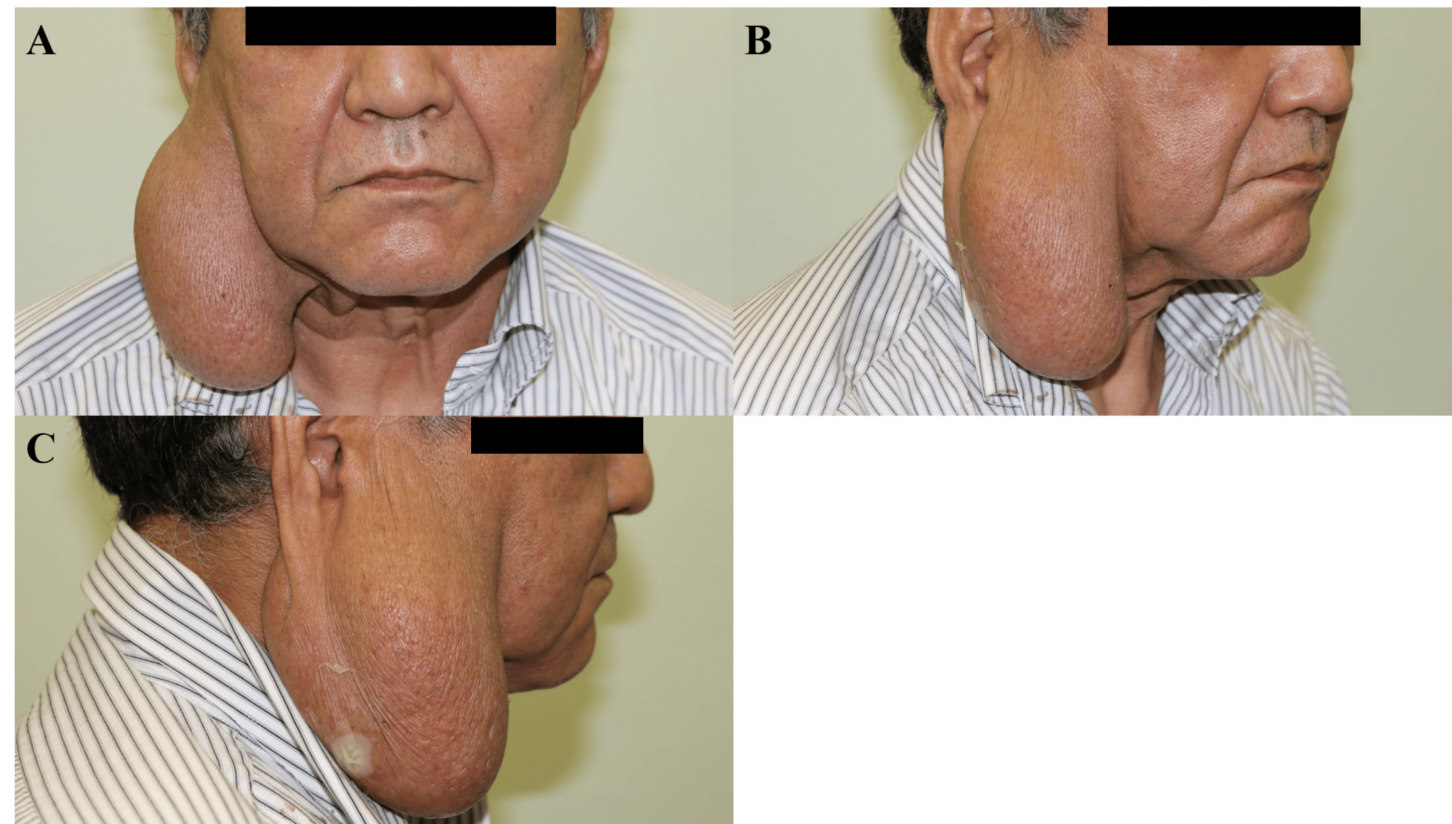
Preoperative clinical presentation Frontal (A), oblique (B), and lateral (C) views showing a large mass in the right parotid region, with visible facial asymmetry, earlobe ptosis, and distortion of anatomical contours.

Physical examination revealed a solitary, well-defined, firm, nontender, superficial parotid mass with prominent subcutaneous projection, measuring approximately 9×6.5 cm. The lesion was immobile in all directions due to its origin in the superficial lobe of the parotid gland and its substantial size. However, it was not adherent to the overlying skin, which was intact, non-erythematous, and freely mobile. The mass had smooth surface contours and clearly palpable margins. Although it created a prominent subcutaneous bulge, the lesion itself did not originate from subcutaneous tissue. Secondary features included marked facial asymmetry and right earlobe ptosis. There was no cervical lymphadenopathy, and facial nerve function was intact, corresponding to House-Brackmann Grade I (Figure 1).

**Figure 2:**
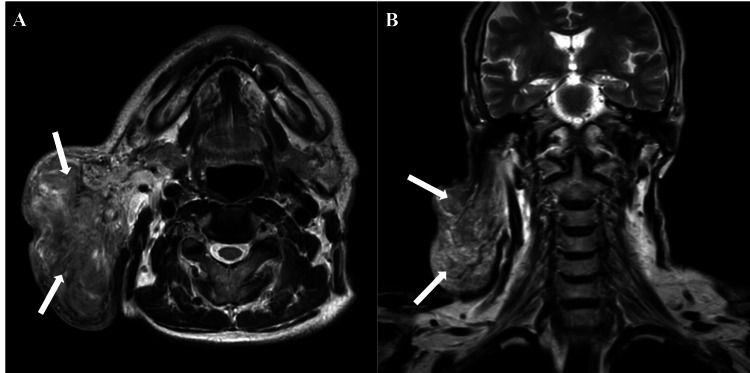
MRI findings Axial (A) and coronal (B) T2-weighted images showing a homogeneous, well-defined mass in the right parotid gland extending posteriorly but without deep tissue invasion or neurovascular involvement. MRI: magnetic resonance imaging

**Figure 3:**
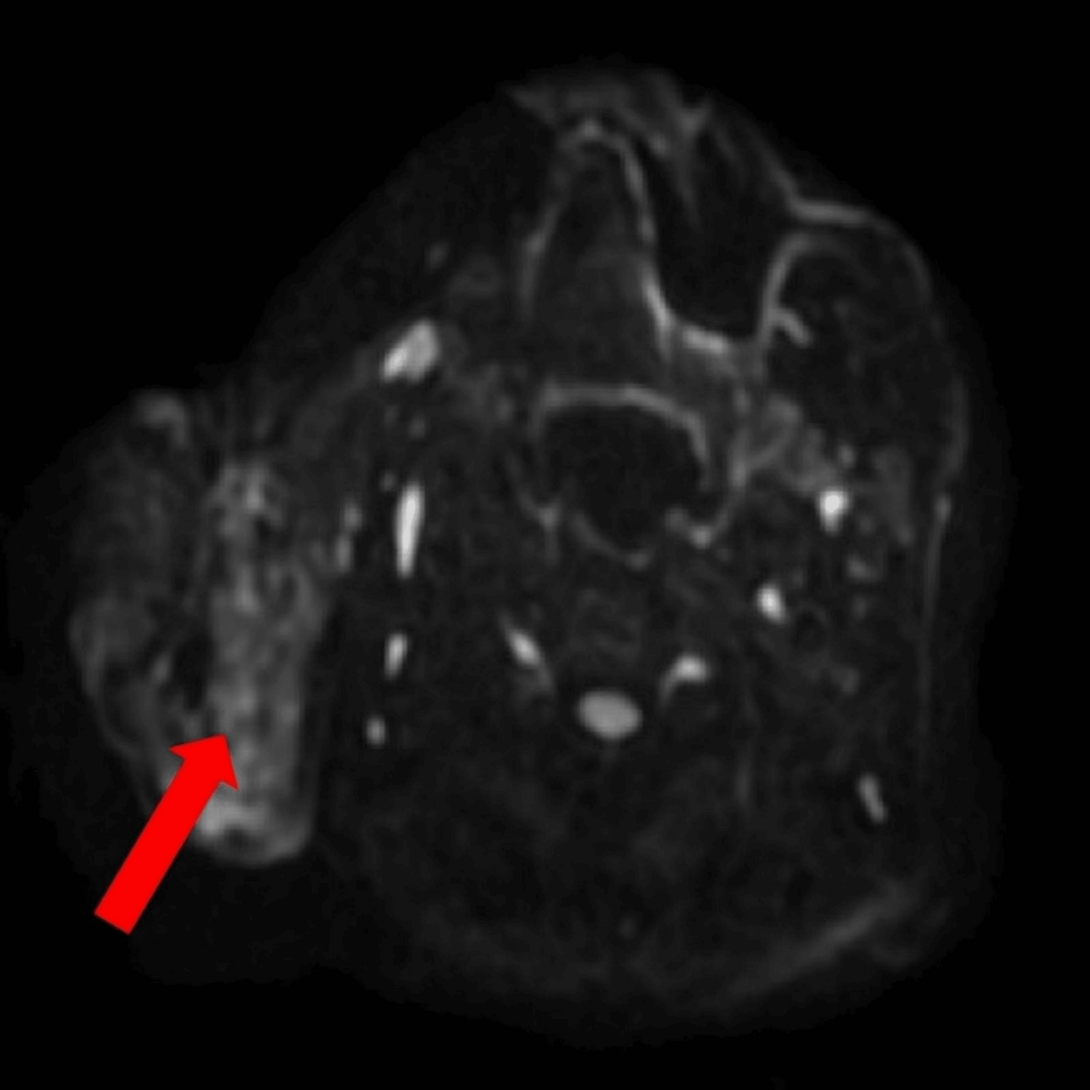
DWI Axial DWI demonstrating a hyperintense lesion in the right parotid gland (red arrow), consistent with an inflammatory process. No cystic, necrotic, or diffusion-restrictive features are noted. DWI: diffusion-weighted imaging

MRI confirmed a well-circumscribed lesion localized to the superficial lobe of the right parotid gland, without evidence of extension into the deep lobe or parapharyngeal space. There was no medialization of the oropharynx, and the oropharyngeal mucosa appeared intact. The lesion was homogeneous on T2-weighted sequences, without signs of cystic degeneration, necrosis, or lobulation (Figure 2). DWI demonstrated a hyperintense signal within the lesion, with no evidence of restricted diffusion, findings consistent with a dense inflammatory infiltrate rather than neoplastic or cystic pathology (Figure 3).

Clinically and radiologically, the lesion appeared grossly well-defined with smooth contours and discrete margins. However, histologic analysis later revealed diffuse eosinophilic infiltration into adjacent fibroadipose tissue, indicating microscopically ill-defined margins, a characteristic feature of KD.

Preoperative laboratory investigations revealed persistent peripheral eosinophilia, with an absolute eosinophil count above 0.8×103/L. Although serum IgE was not tested during this admission, prior laboratory results reportedly had elevated IgE levels. Renal function remained normal, with no proteinuria or elevated urea/creatinine. A summary of hematologic, biochemical, and inflammatory markers is provided in Table 1, illustrating a postoperative decline in eosinophil count following lesion excision.

**Table 1:** Laboratory investigation results Selected hematologic, biochemical, and inflammatory markers from the initial evaluation through postoperative recovery. Eosinophil levels decreased substantially following surgical excision. ALB: albumin; ALP: alkaline phosphatase; ALT: alanine aminotransferase; AST: aspartate aminotransferase; Band cells: immature neutrophils; Basophils: basophilic granulocytes; Cl: chloride; CRE: creatinine; CRP: C-reactive protein; eGFR: estimated glomerular filtration rate; Eosinophils: eosinophilic granulocytes; GLU: glucose; Hb: hemoglobin; Ht: hematocrit; K: potassium; LD: lactate dehydrogenase; MCH: mean corpuscular hemoglobin; MCHC: mean corpuscular hemoglobin concentration; MCV: mean corpuscular volume; Monocytes: mononuclear phagocytes; Na: sodium; PLT: platelet count; RBC: red blood cell count; T BIL: total bilirubin; TP: total protein; UN: urea nitrogen; WBC: white blood cell count; γ-GT: gamma-glutamyl transferase

Parameter	Initial visit	Preoperative evaluation	Postoperative day 4	Postoperative day 20	Reference range	Units
WBC	7	6.7	9.7	5.8	3.3-8.6	×10³/μL
RBC	4.39	4.71	4.56	4.48	4.35-5.55	×10⁶/μL
Hb	14	14.9	14.3	13.9	13.7-16.8	g/dL
Ht	41.3	43.4	43.2	41.4	40.7-50.1	%
MCV	94.1	92.1	94.7	92.4	83.6-98.2	fL
MCH	31.9	31.6	31.4	31	27.5-33.2	pg
MCHC	33.9	34.3	33.1	33.6	31.7-35.3	g/dL
PLT	375	387	354	581	158-348	×10³/μL
Neutrophils	-	-	70.3	71.5	40.7-77	%
Band cells	2	0.5	-	-	0.5-6.5	%
Lymphocytes	16	17	14	20.8	38-74	%
Monocytes	7	4.5	6.6	5	2-10	%
Eosinophils	18	12	7.2	1.7	2-10	%
Basophils	2	1.5	1.2	1	0-8.5	%
TP	6.5	6.9	6.8	7.4	6.6-8.1	g/dL
ALB	4.2	4.5	-	-	3.5-5	g/dL
GLU	97	-	-	-	73-109	mg/dL
UN	11	15	7	10	8-20	mg/dL
CRE	0.69	0.65	0.65	0.66	0.65-1.07	mg/dL
eGFR	87.5	93.4	93.4	91.8	≥60	mL/min/1.73 m²
T BIL	0.6	0.5	0.6	0.4	0.4-1.5	mg/dL
Na	141	142	143	142	138-145	mmol/L
K	4.5	4.4	4.7	4.7	3.6-4.8	mmol/L
Cl	104	106	105	105	101-108	mmol/L
AST	20	17	25	22	13-30	U/L
ALT	13	15	22	34	10-42	U/L
ALP	60	56	58	75	38-113	U/L
LD	223	209	240	225	124-222	U/L
γ-GT	18	24	20	77	13-64	U/L
CRP	-	<0.10	0.61	<0.10	0-0.14	mg/dL

**Figure 4:**
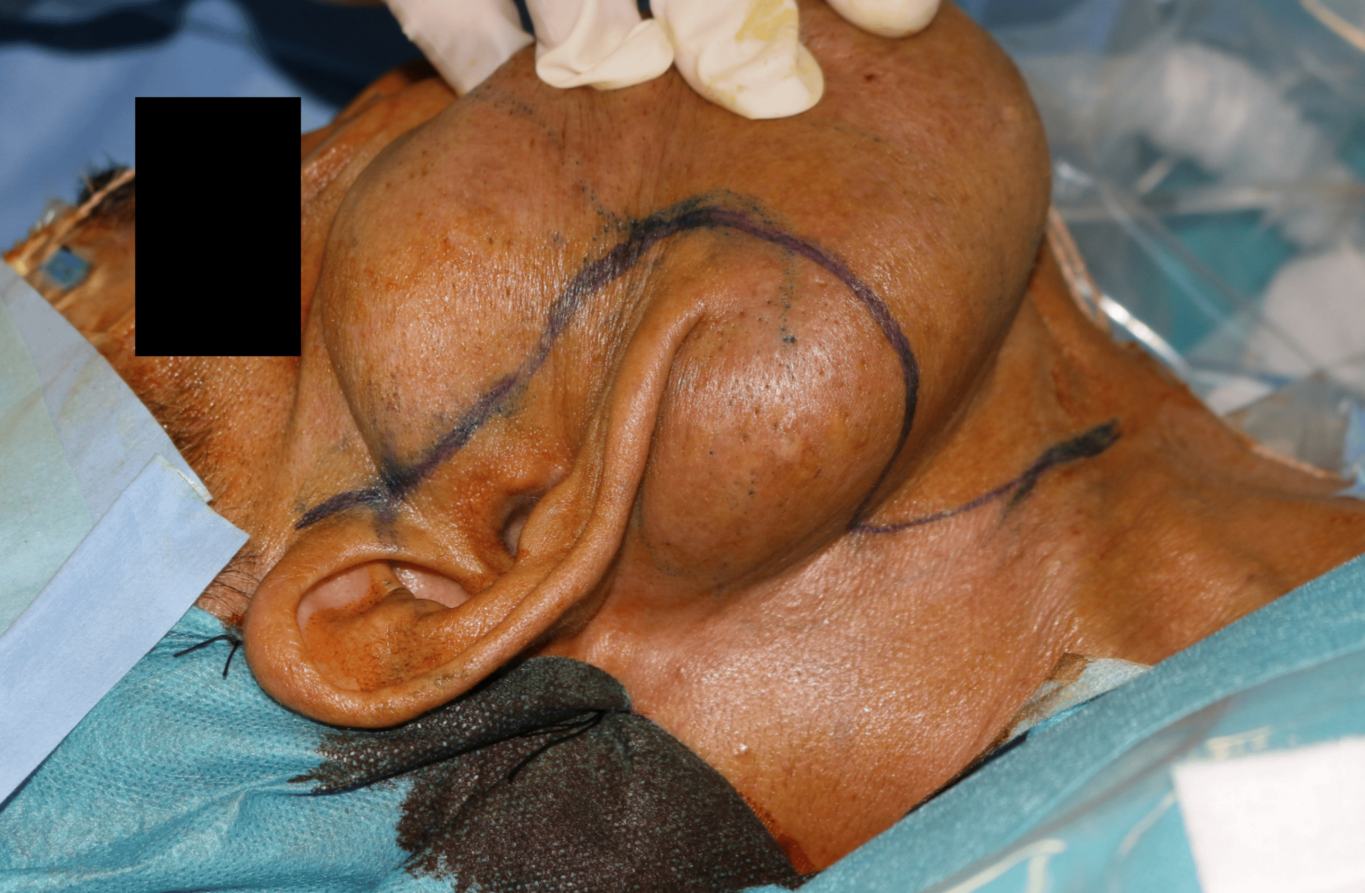
Surgical planning and incision design Preoperative markings of a modified rhytidectomy incision extending from the temporal hairline along the preauricular crease, around the earlobe, and into the posterior scalp. The cervical extension follows relaxed skin tension lines in an S-shaped pattern.

Given the lesion's chronicity, size, and aesthetic implications, the patient elected to undergo surgical excision. A facelift-type (modified rhytidectomy) incision was planned to maximize exposure while minimizing visible scarring (Figure 4).

**Figure 5:**
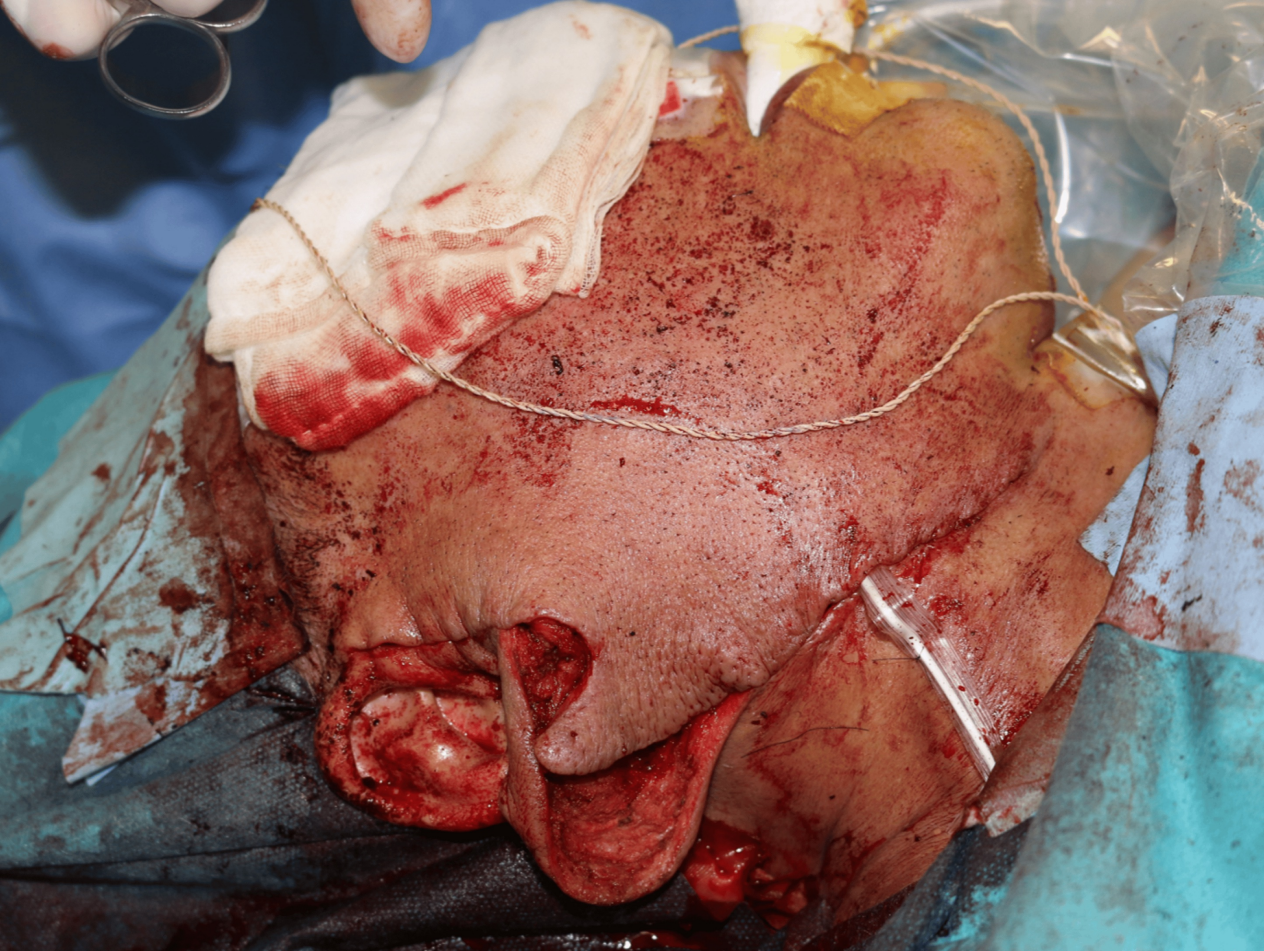
Intraoperative surgical approach Modified rhytidectomy incision with elevation of a subcutaneous skin flap, preserving the vascularized overlying skin. Dissection proceeded in the intermediate fat layer, avoiding unnecessary SMAS manipulation. SMAS: superficial musculoaponeurotic system

A posteriorly based facelift-type incision was made, curving around the ear and extending into the posterior hairline. Dissection proceeded within the subcutaneous plane, superficial to the superficial musculoaponeurotic system (SMAS), which was preserved and not elevated. The lesion was localized to the inferior pole of the superficial lobe of the right parotid gland. It extended anteriorly but did not involve the deep lobe or masseter muscle. A superficial parotidectomy was performed, and facial nerve branches were visualized and preserved without the need for deep dissection (Figure 5).

**Figure 6:**
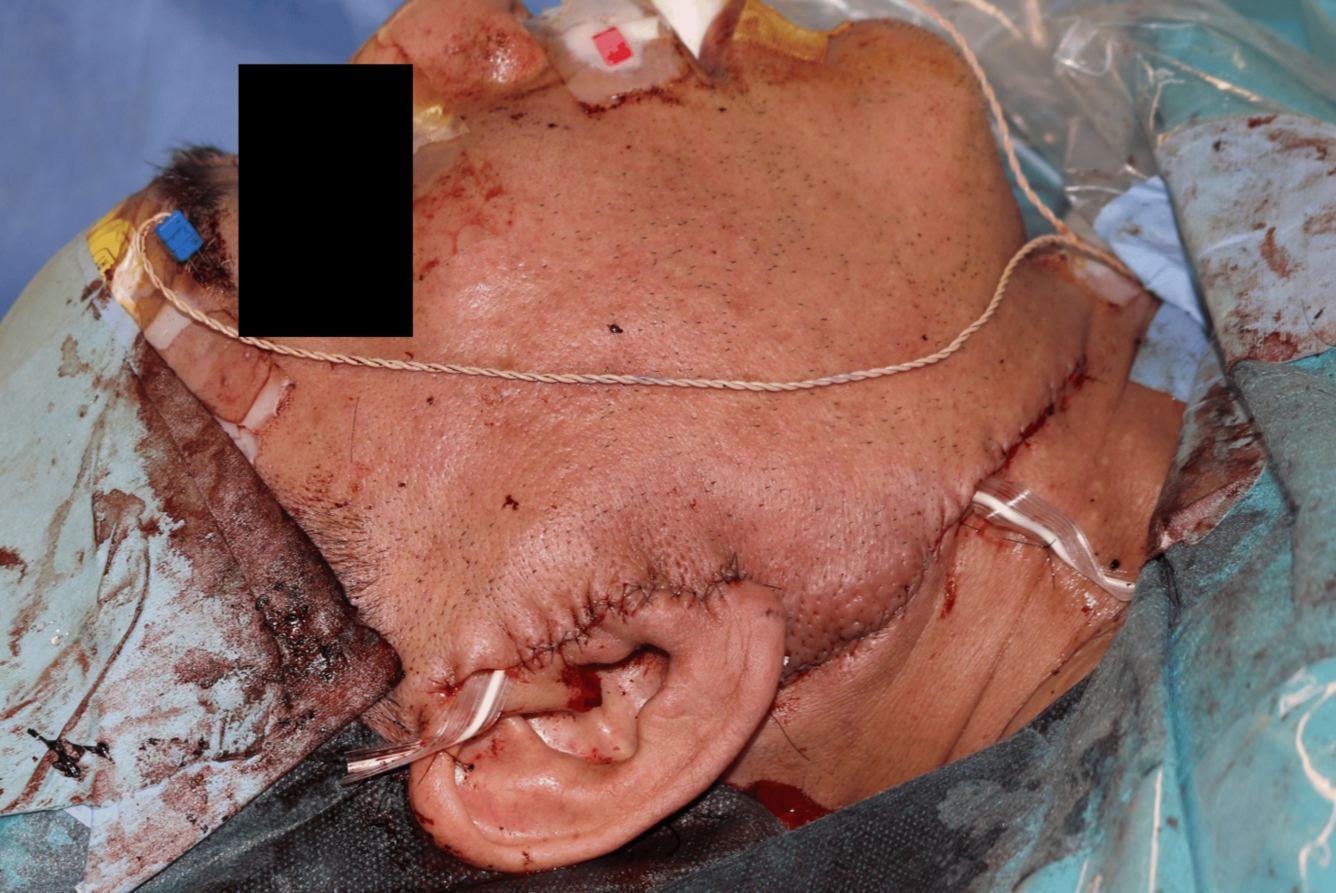
Earlobe reconstruction after tumor excision Intraoperative image showing correction of earlobe ptosis using a posteriorly based advancement flap aligned to the contralateral side.

The overlying skin was preserved, with no dermal tethering or invasion observed. Dissection proceeded along a distinct connective tissue plane between the lesion and subcutaneous tissue. SMAS suspension or repositioning was not performed. Earlobe ptosis was corrected using a posteriorly based subcutaneous advancement flap anchored to the mastoid periosteum (Figure 6).

**Figure 7:**
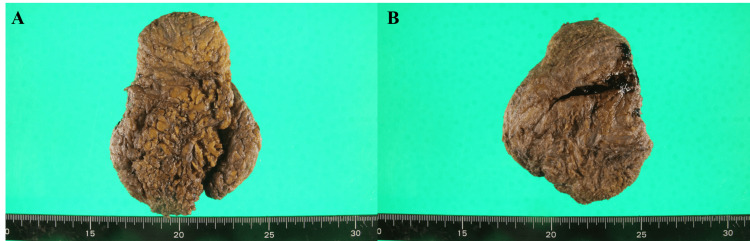
Gross pathology specimen (A) Entire excised mass measuring 117×85×43 mm. (B) Cross-section showing a mottled yellow-white surface consistent with fibrosis and inflammation characteristic of KD. KD: Kimura disease

The excised specimen measured 11.7×8.5×4.3 cm and was submitted for gross and microscopic analysis (Figure 7).

**Figure 8:**
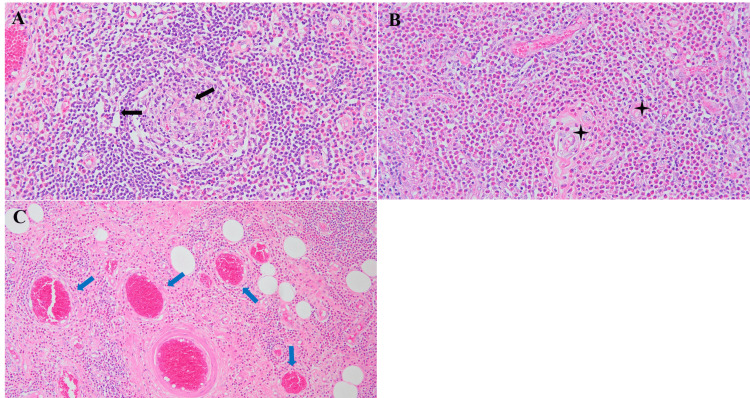
Histopathologic features of KD (A) Low-power view (H&E staining magnification ×100) showing follicular hyperplasia and preserved nodal architecture (black arrowheads). (B) Medium-power view (H&E staining magnification ×200) with dense eosinophilic infiltrates and eosinophilic abscesses (asterisk). (C) Low-power view (H&E staining magnification ×100) revealing vascular proliferation and postcapillary venule hyperplasia (blue arrowheads). KD: Kimura disease

Microscopic evaluation revealed florid lymphoid follicular hyperplasia, dense eosinophilic infiltration, eosinophilic microabscesses, and marked postcapillary venule proliferation. The lesion demonstrated diffuse infiltration into surrounding fibroadipose tissue, confirming microscopically ill-defined borders despite gross circumscription. No neoplastic epithelium, mitotic figures, or atypical lymphoid cells were observed. Given the prior histologic diagnosis and characteristic morphology, immunohistochemical (IHC) staining was not repeated. Warthin's tumor, lymphoma, ALHE, and IgG4-related disease were excluded based on classic histologic features (Figure 8).

**Figure 9:**
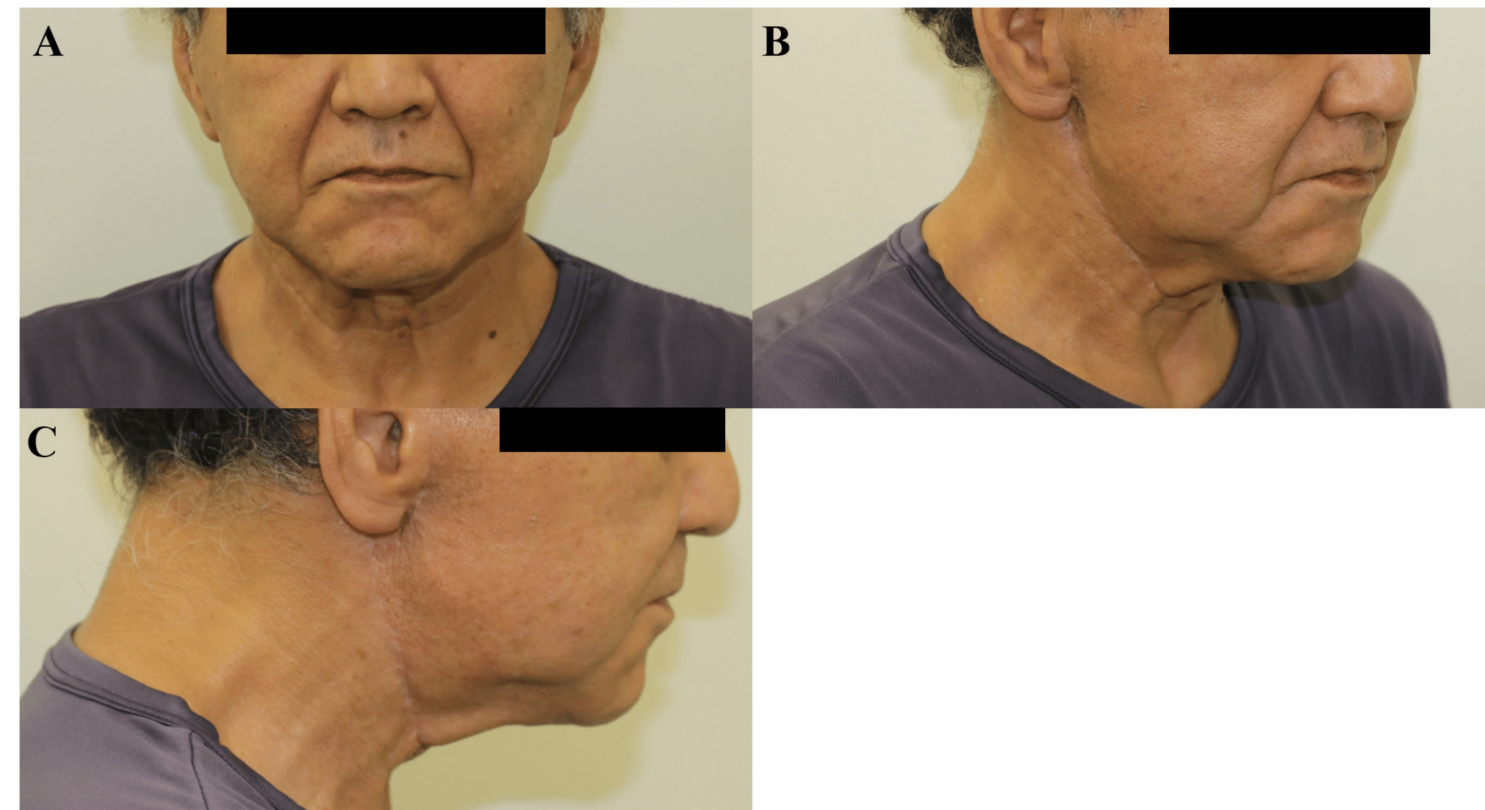
Six-month postoperative result Frontal (A), oblique (B), and lateral (C) views showing restored facial symmetry, corrected earlobe contour, and inconspicuous postoperative scarring. No recurrence is evident.

No adjuvant medical therapy, including corticosteroids, was administered postoperatively. Given the complete excision, absence of systemic symptoms, and stable laboratory parameters, the patient was managed conservatively with serial laboratory monitoring and annual MRI. Facial symmetry was fully restored, and House-Brackmann Grade I function was maintained throughout recovery. At the six-month follow-up, the patient remained asymptomatic with no radiologic or clinical evidence of recurrence and reported high satisfaction with both functional and cosmetic outcomes (Figure 9).

## Discussion

KD is an uncommon, chronic inflammatory lymphoproliferative disorder of unclear etiology. Reports by Park et al. [11] and Lee et al. [12] indicate that most KD lesions range from 1 to 8 cm in diameter, with average sizes between 4 and 6 cm. Consequently, lesions exceeding 9-10 cm may be classified as "giant" and are exceedingly rare. Case reports of unusually large lesions, such as a 13×7×6.5 cm auricular mass and a 7 cm frontal mass, illustrate the challenges of managing KD in cosmetically sensitive areas [13,14].

The discordance between grossly well-defined appearance and microscopically ill-defined margins is a hallmark of KD, reflecting its infiltrative inflammatory behavior. This feature distinguishes KD from benign salivary neoplasms, which typically display consistent border definitions across clinical, radiologic, and histologic evaluations.

The differential diagnosis of a well-circumscribed parotid mass includes benign tumors such as Warthin's tumor, pleomorphic adenoma, and lymphoepithelial cysts. In our case, these were effectively excluded based on imaging and histology. The lesion exhibited homogeneous signal intensity, lacked cystic or lobulated features on MRI and DWI, and showed no oncocytic epithelium or ductal elements histopathologically. Instead, hallmark features of KD were observed, including lymphoid follicular hyperplasia, dense eosinophilic infiltration, eosinophilic abscesses, and postcapillary venule proliferation.

Although FNAC is commonly used to evaluate parotid lesions, it is generally insufficient for diagnosing KD, as it lacks the architectural detail required to identify eosinophilic microabscesses, follicular hyperplasia, and vascular proliferation [9]. Excisional biopsy remains the preferred diagnostic method in suspected KD cases, particularly when architectural preservation is essential.

KD may also mimic other inflammatory conditions such as ALHE and IgG4-related disease. ALHE typically presents as superficial dermal papules or nodules and lacks the florid follicular hyperplasia seen in KD; it also features prominent epithelioid endothelial cells and fewer germinal centers. In contrast, IgG4-related disease is often characterized by storiform fibrosis, obliterative phlebitis, and elevated serum IgG4 levels, none of which were observed in our patient. Our histopathology revealed preserved lymph node architecture, dense eosinophilic infiltration with abscess formation, and postcapillary venule hyperplasia, findings pathognomonic for KD. Immunohistochemistry was therefore not necessary in this case, although it may be helpful in diagnostically ambiguous presentations.

Renal involvement is among the most significant systemic manifestations of KD, reported in 12-20% of patients, and many develop nephrotic-range proteinuria responsive to corticosteroids [7,15]. The proposed mechanism involves IgE-mediated immune complex deposition and eosinophilic infiltration of the glomeruli; however, some biopsy specimens lack eosinophils, suggesting additional immune pathways may be involved [7]. In our case, renal function remained stable, and no proteinuria or other signs of nephropathy were noted during the perioperative period.

To aid clinical recognition, we compiled a summary of key diagnostic features, including history, physical findings, laboratory data, imaging, and histopathology, in Table 2. Peripheral eosinophilia and elevated serum IgE levels are characteristic laboratory findings supportive of KD diagnosis [16].

**Table 2:** Summary of the diagnostic components that supported the final diagnosis of KD Peripheral eosinophilia, characteristic histopathology, and radiologic exclusion of neoplasm were key determinants. Supporting references correspond to full citations in the manuscript's reference list. KD: Kimura disease; MRI: magnetic resonance imaging; DWI: diffusion-weighted imaging

Category	Findings	Diagnostic relevance	Supporting literature
Patient history	A 66-year-old Japanese man with an 18-year history of a progressively enlarging right parotid mass	Chronicity and recurrence suggest an inflammatory rather than a neoplastic etiology	[4,12]
Physical examination	Firm, well-defined, immobile, nontender, 9×6.5 cm mass; intact skin; earlobe ptosis; no lymphadenopathy; House-Brackmann Grade I facial nerve function	Suggests superficial lobe involvement without nerve compromise	[11,17]
Laboratory findings	Documented peripheral eosinophilia; prior elevated IgE (not repeated during current presentation)	Strongly supportive of an eosinophilic inflammatory process such as KD	[5,6,16]
Imaging (MRI/DWI)	Well-circumscribed superficial lesion; no cystic, necrotic, or infiltrative features; no deep lobe extension	Helps exclude Warthin's tumor, pleomorphic adenoma, and malignant parotid neoplasms	[11,17]
Histopathology	Lymphoid follicular hyperplasia, eosinophilic infiltration and abscesses, postcapillary venule proliferation	Pathognomonic features of KD	[5,6,16]
Final diagnosis	KD based on clinicopathologic correlation and prior histologic confirmation	Supported by chronic course, radiologic exclusion of neoplasm, and characteristic histology	[4,12,13]

Corticosteroids remain a cornerstone of systemic KD management, particularly in cases with nephrotic syndrome or incomplete resection. Although corticosteroids induce symptomatic relief in most patients, recurrence is common upon dose tapering, and long-term use carries significant side effects [3,5]. Immunosuppressive agents such as cyclosporine and leflunomide have demonstrated variable efficacy but require close monitoring for toxicity [6]. Radiotherapy has shown efficacy for inoperable or recurrent lesions; however, concerns regarding tissue fibrosis and long-term risks limit its use in younger patients [5,10].

Recent advances include biologic agents targeting Th2 cytokines, such as dupilumab (anti-IL-4Rα), mepolizumab (anti-IL-5), and omalizumab (anti-IgE). While early results from case series are promising, further research is required to validate their efficacy and long-term safety [10].

No systemic corticosteroids or immunomodulatory therapies were initiated postoperatively in our patient, as the lesion was completely resected and the patient remained asymptomatic without systemic manifestations. A structured surveillance strategy was implemented, including biannual clinical examinations, annual MRI scans, and periodic blood tests to monitor eosinophil counts and renal function. Although our patient remains recurrence-free at six months, we recommend close monitoring during the first two years, when recurrence is most likely. Published studies support clinical follow-up with eosinophil counts and imaging every 6-12 months for two years, followed by annual surveillance through year 5 [10,12].

From a surgical perspective, complete excision in the parotid region presents unique technical and aesthetic challenges. Traditional parotid incisions, such as the modified Blair approach, offer excellent access but often leave conspicuous scars. Recent comparative reviews favor facelift (rhytidectomy-type) incisions over Blair incisions for superior aesthetic outcomes without compromising surgical access [18,19]. In this case, a modified rhytidectomy incision allowed wide exposure while concealing scars behind the auricle and along relaxed skin tension lines, advantageous given the lesion's size and superficial location.

Facial nerve preservation was achieved without intraoperative nerve monitoring by carefully following anatomical landmarks and remaining within the superficial parotid plane. The SMAS layer was preserved and not elevated, preventing postoperative facial asymmetry. Correction of earlobe ptosis was performed using a posterior-pivot V-flap anchored subcutaneously to the mastoid periosteum, providing durable contour restoration and minimizing wound tension. These technical refinements contributed to excellent cosmetic and functional outcomes without complications.

This case highlights the importance of integrating aesthetic surgical principles into the treatment of benign inflammatory lesions. The modified rhytidectomy incision and associated tissue-handling strategies not only facilitated complete tumor removal but also preserved appearance in this highly visible anatomical region. As KD is prone to recurrence and often affects younger patients, such techniques may improve long-term quality of life and reduce the need for revision procedures.

This report is not without limitations. First, serum IgE levels, typically elevated in KD, were not remeasured during this presentation, although they had reportedly been elevated in the past by the initial treating physician. Second, no preoperative FNAC was performed, which may be considered a diagnostic limitation. However, this decision was justified based on the patient's prior histological confirmation of KD, slow progression, and supportive imaging findings. Third, while histopathologic findings were typical, no IHC staining was performed, although key mimics were effectively excluded based on morphology. Finally, although our patient is recurrence-free at six months, we recommend clinical evaluation with eosinophil counts and radiological surveillance (ultrasound or MRI) every 6-12 months during the first two years, when the recurrence risk is highest, followed by annual monitoring through year 5. This approach aligns with reported relapse patterns, which typically occur within the first one to two years following treatment [7,12].

## Conclusions

Giant KD involving the parotid region is exceptionally rare, posing significant diagnostic and surgical challenges. This case demonstrates that complete lesion excision, preservation of facial nerve function, and optimal aesthetic outcomes can be achieved using a modified rhytidectomy (facelift-type) incision. Technical refinements, including a posterior-pivot V-flap for the correction of earlobe ptosis and subcutaneous tissue anchoring to the mastoid periosteum, facilitated the restoration of facial symmetry, minimized contour deformity, and resulted in discreet scarring. These strategies underscore the value of incorporating aesthetic surgical principles into the management of benign inflammatory lesions, especially in cosmetically sensitive regions.

Given the known risk of delayed recurrence, particularly in lesions larger than 9 cm, we recommend structured postoperative surveillance comprising biannual clinical evaluations and annual imaging for the first two years, followed by annual monitoring thereafter. Although our patient remains recurrence-free at six months, ongoing long-term follow-up is essential to confirm sustained disease control. This report offers a reproducible, aesthetics-focused surgical approach to parotid KD resection and may serve as a practical model for managing similarly large, localized lesions without compromising cosmetic integrity.

## References

[1] Kim HT (1937). Eosinophilic hyperplastic lymphogranuloma, comparison with Mikulicz's disease. Chin Med J.

[2] Kimura T, Yoshimura S, Ishikawa E (1948). On the unusual granulation combined with hyperplastic changes of lymphatic tissues. Trans Soc Pathol Jpn.

[3] Yuen HW, Goh YH, Low WK, Lim-Tan SK (2005). Kimura's disease: a diagnostic and therapeutic challenge. Singapore Med J.

[4] Li X, Wang J, Li H, Zhang M (2019). Misdiagnosed recurrent multiple Kimura's disease: a case report and review of the literature. Mol Clin Oncol.

[5] Kim WJ, Kim HK (2022). Current concepts of Kimura disease: pathophysiology and evolution of treatment. Arch Craniofac Surg.

[6] Katagiri K, Itami S, Hatano Y, Yamaguchi T, Takayasu S (1997). In vivo expression of IL-4, IL-5, IL-13 and IFN-gamma mRNAs in peripheral blood mononuclear cells and effect of cyclosporin A in a patient with Kimura's disease. Br J Dermatol.

[7] Loymans RJ, Berend K, Abreu de Martinez VG, Florquin S, de Vries AP (2011). Nephrotic syndrome in Kimura's disease: apropos a case of the glomerular tip lesion in an African-Caribbean male. NDT Plus.

[8] Shaikh M, Garg P, Sharma P, Khera P (2019). MRI evaluation of Kimura's disease with emphasis on diffusion weighted imaging and enhancement characteristics. Indian J Radiol Imaging.

[9] Nga ME (2024). Pitfalls in lymph node fine needle aspiration cytology. Acta Cytol.

[10] Bellinato F, Mastrosimini MG, Querzoli G, Gisondi P, Girolomoni G (2022). Dupilumab for recalcitrant Kimura disease. Dermatol Ther.

[11] Park SW, Kim HJ, Sung KJ, Lee JH, Park IS (2012). Kimura disease: CT and MR imaging findings. AJNR Am J Neuroradiol.

[12] Lee CC, Feng IJ, Chen YT (2022). Treatment algorithm for Kimura's disease: a systematic review and meta-analysis of treatment modalities and prognostic predictors. Int J Surg.

[13] Sakanoue M, Matsushita S, Kawai K, Kanekura T (2014). A case of Kimura's disease as giant pedunculated tumors. Indian J Dermatol.

[14] Yoshida T, Nishimura K, Waki D, Tanaka N, Murabe H, Yokota T (2022). Clinical images: giant mass on the forehead in Kimura disease. ACR Open Rheumatol.

[15] Nakahara C, Wada T, Kusakari J (2000). Steroid-sensitive nephrotic syndrome associated with Kimura disease. Pediatr Nephrol.

[16] Ohta N, Okazaki S, Fukase S, Akatsuka N, Aoyagi M, Yamakawa M (2007). Serum concentrations of eosinophil cationic protein and eosinophils of patients with Kimura's disease. Allergol Int.

[17] Zhao F, Zhou M, Mao A, Zhang Y, Chen Y (2024). Kimura disease: a detailed analysis of clinical and radiological manifestations in a retrospective case series. J Inflamm Res.

[18] Lee YC, Liao WC, Yang SW (2021). Systematic review and meta-analysis of modified facelift incision versus modified Blair incision in parotidectomy. Sci Rep.

[19] Yin S, Han Y, Liu Y (2022). Comparison of various surgical incisions in parotidectomy: a systematic review and network meta-analysis. Front Oncol.

